# Fluocinolone acetonide 0.18-mg implant for treatment of recurrent inflammation due to non-infectious uveitis: a case series of 15 patients

**DOI:** 10.1186/s12348-024-00427-9

**Published:** 2024-09-19

**Authors:** Robert A. Sisk, Daniel F. Kiernan, David Almeida, Anton M. Kolomeyer, David Eichenbaum, John W. Kitchens

**Affiliations:** 1 Cincinnati Eye Institute, 1945 CEI Drive, Cincinnati, OH 45242 USA; 2 Retina Partners of Florida, Lakeland, FL USA; 3 Erie Retinal Surgery, Erie, PA USA; 4 NJ Retina, New Providence, NJ USA; 5 Scheie Eye Institute, Department of Ophthalmology, University of Pennsylvania, Philadelphia, PA USA; 6 Retina Vitreous Associates of Florida, St. Petersburg, FL USA; 7 Retina Associates of Kentucky, Lexington, KY USA

**Keywords:** Non-infectious uveitis, Macular edema, Fluocinolone acetonide, Intravitreal steroid injection, Vitrectomy, Postoperative ocular inflammation

## Abstract

**Introduction:**

Uncontrolled non-infectious uveitis affecting the posterior segment (NIU-PS) can lead to vision loss due to repeated bouts of inflammation and consequent tissue damage. Patients with chronic NIU-PS who experience recurrent uveitis after being treated with systemic and short-acting local corticosteroids may benefit from the sustained-release 0.18-mg fluocinolone acetonide implant (FAi).

**Methods:**

In this case series, 18 eyes with chronic, recurrent NIU-PS and cystoid macular edema (CME) treated with the 0.18-mg FAi were analyzed retrospectively. Data on patient demographics, clinical history, previous and concomitant treatments for uveitis recurrence, time to and number of uveitis recurrences, intraocular pressure (IOP), central subfield thickness (CST), and visual acuity (VA) were collected and summarized.

**Results:**

A majority of patients (14/15 [93%]) had a history of ocular surgery, largely cataract extraction, and all developed chronic and recurrent NIU-PS and CME. At baseline, patients had a mean age of 72 years (range: 46 to 93), were 53% male, and had a mean duration of NIU-PS of 3 years (range: 1 to 19). Patients were followed for an average of 16.5 months (range: 2 to 42.5 months) post FAi. Eleven of the 18 eyes (61%) had ≥ 5 recurrences of uveitis since diagnosis, with an average time to recurrence of approximately 12 weeks (range: 1 to 27). All eyes treated with the 0.18-mg FAi showed reduced NIU-PS recurrence and visual and anatomical improvement, as measured by VA and CST, respectively. Two eyes had an IOP elevation that was managed with topical therapy, and one eye was treated with topical prednisolone for additional inflammation management. Two eyes required adjunct therapy with short-acting intravitreal corticosteroids at 7 and 16 weeks for NIU-PS recurrence after 0.18-mg FAi insertion.

**Conclusion:**

After receiving the 0.18-mg FAi, eyes with uncontrolled NIU-PS had sustained resolution of CME and inflammation with limited need for supplementary steroid drops or injections and minimal steroid class-specific adverse effects; none required incisional IOP-lowering surgery.

**Supplementary Information:**

The online version contains supplementary material available at 10.1186/s12348-024-00427-9.

## Background

Chronic uveitis can lead to ocular tissue damage and vision loss due to repeated bouts of inflammation [1]. Uveitis is a major cause of visual morbidity among working-age adults (age 20 to 65 years). A study conducted by Durrani et al. showed prolonged visual loss in two-thirds of patients with non-infectious uveitis, with 22% meeting the criteria for legal blindness [2, 3]. Uveitis is typically classified according to primary location of inflammation along the uveal tract [4, 5]. Intermediate, pan-, and posterior uveitis, while less common than anterior uveitis, often require either systemic or intraocular corticosteroid treatment to effectively manage inflammation [1, 6]. Posterior uveitis can affect the choroid and/or the retina, and is the second most common form of uveitis [4, 6], with 67–90% of posterior uveitis cases being non-infectious [7–10]. Prior ocular surgery is another common cause of acute and chronic uveitis accompanied by macular edema [4, 7]. Non-infectious uveitis affecting the posterior segment (NIU-PS), though not a pre-defined clinical term in the literature, is an umbrella term referring to inflammation affecting the back of the eye (encompassing intermediate, posterior, and panuveitis) of non-infectious origin. This includes, but is not limited to, idiopathic causes, autoimmune conditions, or surgical insults that result in recurrent inflammation of the posterior eye segment [4, 7].

NIU-PS is often managed with systemic corticosteroids, but long-term use of high-dose corticosteroids can be associated with systemic and ocular side effects. Intraocular and periocular corticosteroids reduce systemic exposure and incidence of adverse events by providing adequate concentration of drug at the site of inflammation. Long-acting intraocular implants have the potential to provide extended control of disease and decrease patient burden by reducing the need for repeated injections and the frequency of disease flares, resulting in better overall control of inflammation [11, 12].

**Table 1:** Baseline characteristics

Case	Sex (F/M)	Age (years)	NIU diagnosis year^*^	Ocular history	Medical history	Previous ocular surgeries	Previous ocular therapy(s)	Number of recurrences^†^	Time to recurrence^†^
1	M	68	2017	OU: Pathologic myopia, RD, possible glaucoma OD: persistent CME, ERM, macular schisis, macular hole, panuveitis, wound leak, choroidal detachment, and hypotony	HLD, OA, depression, iron deficiency anemia	OD: RD repair with PPV, endolaser, 25% SF6 tamponade, but shortly after, developed recurrent macula-involving RD; repaired with PPV, inferior retinectomy, endolaser, off-label IMI and silicone oil replacement Macular hole repair with PPV, ERM removal, SF6 tamponade, and ILM peeling	OD: STA (Kenalog) injection at time of silicone oil removal, topical difluprednate 0.05% and ketorolac QID, but switched to dorzolamide and brimonidine due to steroid response; topical prednisolone BID, DEX IVI q 10–12 weeks (11 total injections) Wound leak pressure patching with topical atropine and prednisolone	≥ 5	Every 2–12 weeks
2	F	53	2021	OU: Posterior cyclitis and uveitis, CME	Anxiety, depression	OD: Premium cataract surgery with presbyopia correcting IOL (Vivity toric)	OD: Loteprednol etabonate TID and bromfenac gtts QD x 2 weeks after surgery; DEX IVI after diagnosis of uveitis	≥ 1	16 weeks
3	F	84	2021	OU: Myopic degeneration and PVD OS: NIPU/pars planitis	High cholesterol, hypothyroidism, Afib, allergies	N/A	OS: bevacizumab IVI (3 total injections); DEX IVI (3 total injections)	≥ 5	Every 1–17 weeks
4	M	61	2019	OU: Nuclear sclerosis OS: NIPU/posterior uveitis	HTN, CAD	OS: PCIOL, PCO, s/p PPV for epiretinal membrane removal	OS: triamcinolone IVI (Kenalog; 2 total injections), DEX IVI, STA (2 total injections), triamcinolone IVI (Triesence; 2 total injections)	≥ 5	Every 7–24 weeks
5	M	80	2019	OD: Nuclear sclerosis OS: PCIOL; NIPU	HTN, CAD	OS: Corneal transplant; PCIOL	OS: triamcinolone IVI (Triesence), DEX IVI (4 total injections)	≥ 5	Every 12–27 weeks
6	F	93	2022	OU: PVD; POAG indeterminate OD: NIPU/posterior uveitis	hypercholesterolemia, hypotension, GERD	OU: PCIOL	None	0	Not applicable
7	F	71	2021	OU: PVD OS: CME; NIPU/pars planitis	GERD, depression, vertigo (Meniere’s disease)	OU: PCIOL; TORIC IOL OS: pseudophakia s/p YAG	OS: s/p STA (Kenalog 40); DEX IVI	≥ 1	24 weeks
8	M	72	2021	OU: Hypertensive retinopathy OD: PCIOL; NIPU/macular edema OS: Cataract	HTN	OD: PCIOL	OD: DEX IVI (2 total injections)	≥ 2	20 weeks
9	M	75	2021	OU: Pars planitis with macular edema	HTN, gout, arthritis, polymyalgia, prostate cancer, PE, PTSD	OU: PCIOL OD: s/p PPV/F-ax/Focal laser for vitreous hemorrhage secondary to PDR	OD: DEX IVI	≥ 1	24 weeks
10	F	74	2011	OU: Pars planitis; focal laser; NPDR; intermediate AMD pseudophakia; ocular HTN; PVD	HTN; DM; hypercholesterolemia, stroke	OU: PCIOL	OU: PSTK DEX IVI (every 12–22 weeks) Dorzolamide/timolol gtt BID	≥ 20	OU: every 12–22 weeks
11	M	87	OD: 2013 OS: 2012	OU: Panuveitis; POAG; PVD OD: ERM	Type 2 DM, HTN, hypercholesterolemia, BPH	OU: PCIOL; Tube shunt (OD 2010; OS 2017)	OU: Triamcinolone IVI (Triesence;1 injection OD; 2 injections OS); DEX IVI (19 total injections)	≥ 20	OU: Every 12–20 weeks
12	F	68	2019	OS: Cataract surgery with IOL replacement; uveitis continued post-surgery	Diet-controlled HLD	OS: complicated CE/IOL underwent PPV PPL; PPV IOL exchange Gore-Tex Sutured Akreos AO-60 for dislocated IOL	OS: triamcinolone IVI (Triesence)	≥2	Every 7–18 weeks
13	M	74	2020	OS: ocular HTN; dislocated IOL and recurrent CME	N/A	OS: complicated CE and anterior chamber paracentesis for high IOP	OS: topical difluprednate, timolol, brimonidine gtt	≥5	Roughly every 24–26 weeks
14	F	63	2020	OS: Macula-off RD with HM vision	N/A	OS: RD buckle and vitrectomy with laser and gas (twice); CE/IOL, vitrectomy, and membrane peeling	OS: 2 triamcinolone (Triesence) IVIs	≥2	Every 12–16 weeks
15	M	46	2004	OU: CE; Pars planitis (intermediate uveitis); recurring CME; NAION	Suspected of having MS; colonization with *C. difficile* after MTX treatment for NIU-PS	OU: CE	OU: Triamcinolone (Triesence) or DEX IVI (≥ 20 injections); MTX (2016–2018); DEX IVI	≥ 20	IVIs every 12–16 weeks; then DEX implant every 24 weeks

^*^Laboratory investigation for infectious and non-infectious causes of posterior uveitis was performed based upon the location (intermediate, posterior) of uveitis and could include but were not limited to anti-nuclear antibody, antineutrophil cytoplasmic antibody, angiotensin converting enzyme, beta-2 microglobulin, lysozyme, HLA typing, quantiferon gold, *treponema pallidum*, and serologies for *Bartonella henselae*,* Borrelia burgdorferi*, and *Toxoplasma gondii*

^†^Uveitis recurrence was determined by each investigator according to their individual practice patterns, using criteria such as increase in cellular activity or vitreous haze attributable to NIU-PS, deterioration in visual acuity, increase in macular edema as measured by CST, and/or the need for additional anti-inflammatory medications

Afib, atrial fibrillation; AMD, age-related macular degeneration; BID, twice daily; BPH, benign prostatic hyperplasia; CAD, coronary artery disease; CE, cataract extraction; CME, cystoid macular edema; CST, central subfield thickness; DEX IVI, dexamethasone intravitreal implant 0.7 mg; DM, diabetes mellitus; ERM, epiretinal membrane; F-ax, fluid air exchange; GERD, gastroesophageal reflux disease; gtt, eye drop; HLA, human leukocyte antigen; HLD, hyperlipidemia; HM, hand motion; HTN, hypertension; ILM, internal limiting membrane; IMI, intravitreal methotrexate infusion; IVI, intravitreal injection; MS, multiple sclerosis; MTX, methotrexate; NAION, non-arteritic anterior ischemic optic neuropathy; NIPU, noninfectious posterior uveitis; NPDR, nonproliferative diabetic retinopathy; OA, osteoarthritis; OD, right eye; OS, left eye; OU, both eyes; PCIOL, posterior chamber intraocular lens; PCO, posterior capsule opacification; PDR, proliferative diabetic retinopathy; PE, pulmonary embolism; POAG, primary open angle glaucoma; PPL, pars plana lensectomy; PPV, pars plana vitrectomy; PSTK, posterior sub-tenon Kenalog; PTSD, post-traumatic stress disorder, PVD, posterior vitreous detachment; QD, every day; QID, four times a day; RD, retinal detachment; SF6, sulfur hexafluoride gas; s/p, status post; STA, sub-tenon triamcinolone acetonide; TID, three times a day; YAG, yttrium aluminum garnet

Approved by the United States Food and Drug Administration (FDA), the intravitreal fluocinolone acetonide implant (FAi) is indicated for use in patients with chronic NIU-PS [13]. The safety and efficacy of treating NIU-PS with a low-dose, sustained-release 0.18-mg intravitreal FAi has been demonstrated in randomized, sham injection controlled, double-masked, phase 3 clinical trials (ClinicalTrials.gov identifiers: NCT01694186 and NCT02746991) and in multiple individual case reports [11–14]. Herein, we report a series of 18 eyes from 15 patients with recurrent NIU-PS and postoperative uveitic cystoid macular edema (CME) that achieved long-term inflammation control when treated with 0.18-mg FAi.

**Table 2:** Effectiveness and safety outcomes after 0.18-mg FAi insertion

Case	Reason for initiating FAi	Eye(s) that received FAi OD/OS/OU	Prior VA	Last VA	Prior OCT/CST (µm)	Last OCT/CST (µm)	Prior IOP (mmHg)	Last IOP (mmHg)	Recurrence-free length	Follow-up notes
1	Post-operative, persistent CME, panuveitis with anterior involvement, and scleral thinning leading to persistent wound leaking plus hypotony, likely from repeated STA injections	OD	CF	20/125	438	386	24	14	33 months	Patient was continued on topical prednisolone BID after FAi insertion; experienced no further complications.
2	Ongoing posterior cyclitis and uveitis with CME post cataract surgery, uncontrolled with DEX IVI	OD	20/100	20/30	652	288	16	23	21 months	Patient remains stable and recurrence free.
3	Recurrent posterior uveitis uncontrolled by short acting DEX IVI and VEGF IVI	OS	CF at 4 feet	20/70	891	617	20	23	10 months (had the FAi for a total of 28 months)	Patient received 4 more injections with DEX IVI (0.7 mg) roughly every 4 months after FAi insertion for breakthrough inflammation. After the fourth DEX IVI, patient has stabilized and is recurrence free.
4	Recurrent posterior uveitis (uveitis diagnosed prior to surgical intervention)	OS	20/50	20/40	646	268	17	13	13 months	Patient remains stable and recurrence free.
5	Recurrent posterior uveitis (uveitis diagnosed prior to surgical intervention)	OS	20/20 − 2	20/40	435	364	12	15	10 months	Patient remains stable and recurrence free.
6	0.18-mg FAi selected as first line treatment for posterior uveitis (uveitis diagnosed prior to surgical intervention)	OD	20/40	20/25	309	291	24	14	2 months	Patient remains stable and recurrence free.
7	Posterior uveitis recurring after 1 DEX IVI injection (uveitis diagnosed prior to surgical intervention)	OS	20/80 + 2	20/50	618	285	13	18	10 months	Patient remains stable and recurrence free.
8	Posterior uveitis recurring after 2 DEX IVI injections (uveitis diagnosed prior to surgical intervention)	OD	20/70	20/60	470	454	16	28	8 months	Patient remains stable and recurrence free.
9	Posterior uveitis recurring after 1 DEX IVI injection (uveitis diagnosed prior to surgical intervention)	OD	20/60	20/40	284	278	8	25	9 months	Patient remains stable and recurrence free.
10	Recurrent and uncontrolled pars planitis OU (uveitis diagnosed prior to surgical intervention); FAi initiated to treat vitritis/CME	OU	OD: 20/50 OS: 20/200	OD: 20/30 OS: 20/60	OD: 211 OS: 194	OD: 204 OS: 194	OD: 12 OS: 12	OD: 14 OS: 13	≥ 4 months	Patient remains stable with no recurrence of edema.
11	0.18-mg FAi selected OU for recurrent and uncontrolled panuveitis (2019; uveitis diagnosed prior to surgical intervention). DEX IVI administered for recurrence (OD 12/2022; OS 1/2023). Second 0.18-mg FAi inserted OU for recurrent inflammation (2023); FAi initiated to treat vitritis/CME	OU	2019 OD: 20/80 OS: 20/40 40 months post first FAi; 20th DEX IVI OU OD: 20/100 OS: 20/50 2023; 2.5 months post second FAi OD: 20/150 OS: 20/100	OD: 20/80 OS: 20/70	2019 OD: 184 OS: 161 22/2023 OD: 218 OS: 227 2023 OD: 219 OS: 232	OD: 266 OS: 230	2019 OD: 24 OS: 17 22/2023 OD: 8 OS: 15 2023 OD: 7 OS: 11	OD: 12 OS: 10	First 0.18-mg FAi: ≥ 36 months Second 0.18-mg FAi: ≥ 2.5 months	With the first 0.18-mg FAi, the patient was recurrence free for ~ 36 months. After roughly 40 months post-implant, the patient had recurrence and was treated by DEX IVI OU. About 1 month after, the second FAi was inserted. Patient regained stability and has had no recurrence of edema.
12	Post-operative, persistent posterior uveitic CME	OS	20/125	20/40+	510	247	14	15	33 months	Patient developed a persistent steroid response to 0.18-mg FAi 3 months post insertion and was started on topical brimonidine tartrate and brinzolamide ophthalmic suspension BID. Patient regained stability and has been recurrence free. IOL is stable and well-centered.
13	Intermediate uveitis and CME recurrence following dislocated IOL, uncomplicated IOL exchange, and placement of ACIOL	OS	20/40	20/30	379	271	24	20	7 months	Initial response to 0.18-mg FAi was limited, so DEX IVI was added. With time, CME receded. Patient is now controlled with topical therapy.
14	Intermediate uveitis and CME recurrence following macula-off retinal detachment repair after 2 DEX IVI	OS	20/60	20/60	398	265	10	19	6 months	Patient remains stable and has been recurrence free.
15	Recurrent CME secondary to intermediate uveitis uncontrolled with ≥ 20 IVI DEX injections (uveitis diagnosed prior to surgical intervention).	OU	OD: 20/30 OS: 20/30	OD: 20/30 OS: 20/25	OD: 240 OS: 252	OD: 247 OS: 241	OD: 11 OS: 14	OD: 9 OS: 12	12 months	0.18-mg FAi OD complicated by endophthalmitis with full recovery of vision after anterior chamber paracentesis & antibiotics (vancomycin and ceftazidime). Patient remains stable and recurrence free without the need for continued IVI.

ACIOL, anterior chamber intraocular lens; CF, counting fingers; CME, cystoid macular edema; CST, central subfield thickness; DEX IVI, dexamethasone intravitreal implant 0.7 mg; FAi, fluocinolone acetonide 0.18-mg implant; IOL, intraocular lens; IOP, intraocular pressure; IVI, intravitreal injection; OCT, optical coherence tomography; OD, right eye; OS, left eye; OU, both eyes; STA, sub-tenon triamcinolone acetonide; VA, visual acuity; VEGF, vascular endothelial growth factor

## Methods

As available from patient medical records, the following data were collected: sex, age, affected eye(s), medical history, ocular history, time since diagnosis, previous treatments, concomitant treatments, number of recurrences, time to recurrence, intraocular pressure (IOP), central subfield thickness (CST), and visual acuity (VA) as measured by each practitioner. Each retrospective case was conducted in accordance with the tenets of the Declaration of Helsinki and the Health Insurance Portability and Accountability Act of 1996. Consent was requested and obtained from the patients for study inclusion.

Investigators determined recurrence of uveitis according to their individual practice patterns, using criteria such as increase in cellular activity or vitreous haze attributable to NIU-PS, deterioration in visual acuity, increase in macular edema as measured by CST, and/or the need for additional anti-inflammatory medications.

## Results

Eighteen eyes diagnosed with non-infectious uveitis and CME were treated by 6 practitioners with the 0.18-mg FAi during active inflammation (Table 1). At baseline, this population had a mean age of 72 years (range: 46 to 93 years), were 53% male, had a mean follow-up of 16.5 months post-FAi (range: 2 to 42.5 months), and had a mean disease duration of 3 years (range: 1 to 19 years) with uncontrolled uveitis before 0.18-mg FAi administration (Table 1). Individual patient narratives and diagnoses are described in detail in the Supplementary Appendix.

All patients were diagnosed with noninfectious uveitis and CME, and for most (14/15 [93%]), a history of cataract or retinal surgery preceded and/or aggravated the diagnosis. Ten patients received their diagnosis prior to cataract or retinal surgery, and 5 patients were diagnosed with post-operative non-infectious uveitis with CME. Of the 15 patients, 8 patients were diagnosed with posterior uveitis, 3 with intermediate uveitis, 2 with panuveitis (1 of these 2 had anterior segment involvement), 1 with pars planitis, and 1 with posterior cyclitis. All patients underwent a diagnostic workup for their uveitis, which included laboratory investigation (Table 1 legend), clinical examination, and ophthalmic imaging, including slit-lamp and dilated fundus examination, optical coherence tomography (OCT), fluorescein angiography, and fundus autofluorescence. One patient had myopic degeneration and posterior vitreous detachment in both eyes, and the left eye had severe pars planitis, leading to chronic and uncontrolled NIU-PS.

Of the 18 eyes in this series, 11 (61%) had 5 or more recurrences of uveitis since initial diagnosis, with an average time to recurrence around 12 weeks (range: 1 to 27). One eye (case #6) had no recurrences before receiving the 0.18-mg FAi (Table 1). The two most common ocular medications used to manage NIU-PS recurrence before treatment with the 0.18-mg FAi were short-acting intravitreal injection (IVI) corticosteroids, including triamcinolone acetonide (56% for each eye) and dexamethasone implant (78% for each eye). Less common IVI medications used in this case series were off-label bevacizumab (1 eye) and off-label methotrexate (3 eyes) (Table 1). Following treatment with the 0.18-mg FAi, all eyes had improvement in VA and decrease in CST as measured by OCT (Table 2).

Of the 18 eyes, 2 were treated with adjunctive intraocular therapy for NIU-PS recurrence following the 0.18-mg FAi: 1 eye (case #13) received a single dexamethasone IVI 7 weeks after the FAi without the need for additional corticosteroid IVI, and another eye (case #3) received ongoing dexamethasone IVI every 17 weeks after the implant (total of 4 dexamethasone IVI), which represented a substantial reduction in IVI corticosteroid treatment frequency (previously every 4 to 6 weeks). Another eye (case #1) required ongoing topical prednisolone for recurrent anterior segment inflammation. Two eyes (case #11) had uveitis recurrence 36 months after 0.18‑mg FAi; this patient then received dexamethasone IVI in both eyes, followed by a second round of 0.18‑mg FAis in both eyes.

Safety events after treatment with the 0.18-mg FAi were tolerable. Only 1 eye from the case series developed persistent IOP elevation after the FAi was injected, and this eye was treated with topical brimonidine tartrate drops; no eyes required surgical intervention for elevated IOP. One eye developed culture-negative endophthalmitis after FAi insertion, and this eye received intravitreal antibiotics. Six weeks after antibiotics were given, the patient’s VA returned to baseline, and the 0.18-mg FAi inhibited uveitis recurrence for more than a year (case #15) (Table 2).

## Discussion

Eyes that undergo multiple intraocular surgeries are at risk for inflammation-related sequelae, including CME, proliferative vitreoretinopathy, and chronic NIU-PS. The mainstay of treatment to manage NIU-PS is local corticosteroids administered topically or via peri/intraocular injection [15]. However, patients with chronic uveitis are at increased risk of secondary glaucoma and cataract formation due to prolonged intraocular inflammation and corticosteroid use [16]. Patients with uveitis also tend to undergo cataract surgery at a younger age than those without uveitis [17–20] and may be at increased risk of postoperative CME and limited visual recovery [21, 22].

There are three FDA-approved IVI FAi dose formulations in the United States, including the high dose 0.59-mg formulation (RETISERT; Bausch + Lomb) indicated for chronic NIU-PS and 2 lower dose formulations, 0.19-mg (ILUVIEN; Alimera Sciences) for diabetic macular edema, and the 0.18-mg FAi (YUTIQ; Alimera Sciences) for chronic NIU‑PS [23]. In phase 3 clinical trials, the 0.18-mg FAi prevented and/or delayed uveitic recurrences, improved VA, and reduced the need for adjunctive therapy among patients with NIU-PS over 36 months [24, 25]. The 0.18-mg FAi is administered in an office setting and delivers a daily dose of 0.2 µg of fluocinolone acetonide [24]. The 0.59-mg FAi, on the other hand, must be surgically implanted [23, 26]. Injection of the 0.18-mg FAi represents an alternative to repeated treatments with shorter-acting corticosteroids, potentially reducing injection-related risks and overall treatment burden, as well as improving long term outcomes by minimizing inflammation recurrences.

As the use of any intraocular corticosteroid may result in IOP elevation, patients should be routinely monitored. Many patients in this case series received intraocular steroid therapy before placement of the 0.18-mg FAi, making the likelihood of an IOP response more predictable. It is hypothesized that the lower dose of fluocinolone (relative to the 0.59-mg implant) and near zero-order kinetics of the long-acting 0.18-mg FAi may be associated with a relatively low propensity for triggering significant increases in IOP [24, 27].

This case series demonstrates real-world experiences and treatment patterns with the 0.18‑mg FAi in eyes with chronic and/or recurrent NIU-PS. In most of these cases, uveitic inflammation was resolved or decreased in intensity for the duration of the FAi lifespan, without the need for additional IVIs (4 eyes required additional steroid IVI post-FAi to help lower inflammation).

In summation, across various surgeons and presentations, the eyes in this series experienced control of NIU-PS and improved vision after treatment with the 0.18 mg FAi, with no new safety signals. Additional studies should enhance our understanding of outcomes with follow up beyond the expected life of the implant.

## Conclusion

Eyes with chronic and/or recurrent non-infectious uveitis affecting the posterior segment had sustained resolution of CME and inflammation after receiving the 0.18-mg FAi, with no new adverse effects.

## Electronic supplementary material

Below is the link to the electronic supplementary material.

Supplementary Material 1

## Data Availability

All data generated or analyzed during this study are included in this published article (and its supplementary information files).

## Abbreviations

CME: cystoid macular edema
CST: central subfield thickness
FAi: fluocinolone acetonide implant
FDA: Food and Drug Administration
IOP: intraocular pressure
IVI: intravitreal injection
NIU-PS: non-infectious uveitis affecting the posterior segment
OCT: optical coherence tomography
VA: visual acuity
