# Supplementary material for: Fluocinolone acetonide 0.18-mg implant for treatment of recurrent inflammation due to non-infectious uveitis: a case series of 15 patients
Source: J Ophthalmic Inflamm Infect. 2024 Sep 19;14:44. doi: 10.1186/s12348-024-00427-9 (PMC11413272; doi:10.1186/s12348-024-00427-9)
Supplement: Supplementary file 1 — Supplementary Material 1 [file 12348_2024_427_MOESM1_ESM.docx]

**Supplementary Material to**

**Fluocinolone Acetonide 0.18-mg Implant for Treatment of Recurrent Inflammation Due to Non-infectious Uveitis: a Case Series of 15 Patients**

Robert A. Sisk, MD, FACS, FASRS^1^, Daniel F. Kiernan, MD, FACS^2^, David Almeida, MD, MBA, PhD^3^, Anton M. Kolomeyer, MD, PhD^4,5^, David Eichenbaum, MD^6^, John W. Kitchens, MD^7^

*^1^Cincinnati Eye Institute, Cincinnati, OH; ^2^Retina Partners of Florida, Lakeland, FL; ^3^Erie Retinal Surgery, Erie, PA; ^4^NJ Retina, New Providence, NJ; ^5^Scheie Eye Institute, Department of Ophthalmology, University of Pennsylvania, Philadelphia, PA; ^6^Retina Vitreous Associates of Florida, St. Petersburg, FL; ^7^Retina Associates of Kentucky, Lexington, KY*

Corresponding Author:

Robert A. Sisk, MD

Cincinnati Eye Institute

1945 CEI Drive

Cincinnati, OH 45242

[rsisk@cvphealth.com](mailto:rsisk@cvphealth.com)

Fax 513-569-3904

**Patient Case Narratives**

**Robert A. Sisk, MD, FACS, FASRS**

**Case #1**

A 68-year-old male patient with a medical history of depression, iron deficiency anemia, hyperlipidemia, and osteoarthritis presented with macula-sparing retinal detachment of the right eye (OD) in 2017. The patient’s ocular history included pathologic myopia, left eye (OS) retinal detachment repaired by scleral buckling, and suspected glaucoma.

This patient received multiple surgeries including pars plana vitrectomy (PPV), endolaser, and 25% sulfur hexafluoride gas tamponade OD. Two weeks post-surgery, the patient developed recurrent macula-involving retinal detachment OD secondary to vitreous base contraction through exuberant laser retinopexy, creating innumerable inferior lacy retinal holes. The right eye’s retina was repaired with PPV, inferior retinectomy, endolaser, silicone oil placement, and off-label methotrexate infusion. Four months later, the patient’s retina remained attached; however, due to his recurrent and severe cystoid macular edema (CME), visual acuity (VA) OD declined from 20/70 to counting fingers, so sub-tenon triamcinolone acetonide (Kenalog) injection was given at the time of silicone oil removal. The patient was diagnosed with postoperative panuveitis (ocular features included iritis, vitritis, and retinal phlebitis) with CME.

The patient remained stable for 17 months, until CME and mild panuveitis recurred with an epiretinal membrane and macular schisis. Laboratory work-up for causes of infectious and non-infectious uveitis were negative. CME and uveitis persisted despite ongoing anti-inflammatory treatment with topical non-steroidal anti-inflammatory drug (NSAID) and corticosteroid (ketorolac and difluprednate 0.05% four times daily). A steroid response increased the patient’s intraocular pressure (IOP) to 24 mmHg, which was treated with dorzolamide and brimonidine. A third retinal surgery consisting of PPV, epiretinal membrane removal, and internal limiting membrane (ILM) peeling was required.

The patient received ongoing topical prednisolone twice daily to cover anterior segment inflammation. CME and panuveitis persisted, and the patient’s VA worsened to 20/200 over the ensuing 6 weeks. Dexamethasone 0.7 mg intravitreal injection (IVI) was placed, resulting in CME reduction but not elimination. IOP was well controlled with topical antihypertensives. The patient required multiple dexamethasone implants about once every 10 to 12 weeks (11 treatments total) OD, for over 27 months; however, he was still experiencing CME and panuveitis and also developed a persistent wound leak (likely due to scleral thinning from repeated injections), hypotony, and choroidal detachment. The patient’s VA dropped to counting fingers but recovered to 20/125 after successful treatment with pressure patching, prednisolone, and atropine drops. On optical coherence tomography (OCT), central subfield thickness (CST) was 438 μm.

The patient refused systemic treatment options for management of unilateral uveitis. Given the need for chronic intravitreal steroids and concerns about risk for recurrent hypotony with continued injections, he was offered the 0.18-mg fluocinolone acetonide implant (FAi). After administration of dexamethasone to quiet the eye, the 0.18‑mg FAi OD was administered. The patient’s inflammation and CME remained controlled through 33 months, with VA of 20/125, CST of 386 μm, and well-controlled IOP.

- VA, CST, and IOP prior to FAi
  - VA: counting fingers
  - CST: 438 μm
  - IOP: 24 mmHg
- VA, CST, and IOP 33 months after FAi
  - VA: 20/125
  - CST: 386 μm
  - IOP: 14 mmHg

**Daniel F. Kiernan, MD, FACS**

**Case #2**

A 53-year-old female patient with a medical history of anxiety and depression, treated with venlafaxine and alprazolam, presented in 2021 with ongoing non-infectious posterior cyclitis and uveitis with CME following cataract surgery OD and the placement of a toric extended depth of focus (EDOF) lens. Patient complained of pain, redness, blurred vision, and foreign body sensation OD 5 weeks after cataract surgery. Slit lamp and dilated fundus exam showed conjunctival injection, 2+ cell, absent flare, 1+ vitreous cell/vitritis, and macular edema. OCT showed extensive intra- and sub-retinal fluid. Fluorescein angiography showed late petaloid macular leakage. Color fundus photos showed blunting of the foveal light reflex. The patient had a routine course of postoperative steroid and NSAID eye drops (loteprednol etabonate three times a day and bromfenac every day) in 2020. After the diagnosis of postoperative posterior cyclitis and uveitis with CME (no presence of vasculitis), the patient was offered to restart drops, but instead elected to have dexamethasone IVI implant 0.7 mg on 01/28/2021. The first recurrence occurred 4 months later. The 0.18-mg FAi implant was administered on 06/02/2021 during active inflammation. The patient has remained stable and recurrence-free for 21 months post-FAi.

- VA, CST, and IOP prior to FAi
  - VA: 20/100
  - CST: 652 μm
  - IOP: 16 mmHg
- VA, CST, and IOP 21 months after FAi
  - VA: 20/30
  - CST: 288 μm
  - IOP: 23 mmHg

**David Almeida, MD, MBA, PhD**

**Case #3**

An 84-year-old female patient with high cholesterol, hypothyroidism, atrial fibrillation, and allergies presented in 2021 with recurrent non-infectious uveitis affecting the posterior segment (NIU-PS) OS, inadequately controlled with a short-acting dexamethasone 0.7-mg implant and intravitreal vascular endothelial growth factor (VEGF) inhibitors. Spectral domain (SD)-OCT, ultra widefield fluorescein angiography, and color fundus photography were used to characterize diffuse NIU-PS with late leakage at the macula and optic nerve. The patient had an ocular history of myopic degeneration and posterior vitreous detachment (PVD) of both eyes, and shortly after, developed pars planitis and NIU-PS OS. The patient experienced <5 recurrences of uveitis and was treated for recurrence every 1 to 17 weeks with short-acting corticosteroids and anti-VEGFs. Treatments included 3 bevacizumab injections and 3 dexamethasone 0.7 mg implants OS. The long-acting 0.18-mg FAi was placed OS on 12/09/2021 during active inflammation, but the patient still experienced uveitis recurrence roughly every 4 months and received a total of 4 dexamethasone 0.7 mg implants post-FAi. The patient has stabilized after the fourth dexamethasone 0.7 mg implant and remains recurrence free for the last 10 months (total of 28 months of follow-up post FAi).

- VA, CST, and IOP prior to FAi
  - VA: counting fingers at 4 feet
  - CST: 891 μm
  - IOP: 20 mmHg
- VA, CST, and IOP roughly 15 months after FAi (and 4 dexamethasone injections)
  - VA: 20/70
  - CST: 617 μm
  - IOP: 23 mmHg

**Case #4**

A 61-year-old male patient with a medical history of hypertension, coronary artery disease, and nuclear sclerosis in both eyes (OU) presented in 2021 with recurrent NIU-PS OS, present since 2019 and aggravated following cataract surgery with posterior chamber intraocular lens (PCIOL) placement. One month after cataract surgery, the patient developed posterior capsule opacification, and underwent PPV/epimacular membrane removal 2 months later. SD-OCT, ultra widefield fluorescein angiography, and color fundus photography were used in the diagnostic work-up, revealing diffuse NIU-PS and macular edema, characterized by late leakage on angiography.

The patient received an injection of sub-tenon triamcinolone 2 weeks before and 1 month after the PPV. The patient subsequently received 2 rounds of IVI triamcinolone (Kenalog), a single dexamethasone 0.7-mg injection, and 2 rounds of IVI triamcinolone suspension (Triesence) OS. This patient had <5 uveitis recurrences, with frequency of every 7 to 24 weeks. Because short-acting therapies provided incomplete control of uveitis recurrence, the long-acting 0.18-mg FAi was placed OS on 12/31/2021 during active inflammation; since then, the eye has remained stable and free from uveitis recurrences.

- VA, CST, and IOP prior to FAi
  - VA: 20/50
  - CST: 646 μm
  - IOP: 17 mmHg
- VA, CST, and IOP 13 months after FAi
  - VA: 20/40
  - CST: 268 μm
  - IOP: 13 mmHg

**Case #5**

An 80-year-old male patient with a history of hypertension and coronary artery disease was diagnosed with recurrent NIU-PS OS in 2019. The patient’s ocular history included nuclear sclerosis OD, a corneal transplant OS that took place in the 1980s, and cataract surgery with a PCIOL placement in 2017. SD-OCT, ultra widefield fluorescein angiography, and color fundus photography were used to characterize diffuse NIU-PS and inflammation of the macula and optic nerve.

Since 2019, the patient had received IVI triamcinolone (Triesence) and 4 dexamethasone 0.7-mg implants OS. This patient had 5 or more recurrences of uveitis with recurrence every 12 to 27 weeks. On 02/03/2022, the patient received the 0.18-mg FAi OS during active inflammation and has remained stable and recurrence free from uveitis for 10 months.

- VA, CST, and IOP prior to FAi
  - VA: 20/20-2
  - CST: 435 μm
  - IOP: 12 mmHg
- VA, CST, and IOP 10 months after FAi
  - VA: 20/40
  - CST: 364 μm
  - IOP: 15 mmHg

**Case #6**

A 93-year-old female patient with a medical history of hypercholesterolemia, hypotension, and gastroesophageal reflux disease (GERD) had PCIOL OU in 2010 and was diagnosed with recurrent NIU‑PS in 2022. The patient’s ocular history included a PVD and indeterminate primary open angle glaucoma (POAG) OU. In 2022, the patient presented to the retina specialist with NIU-PS OD, diagnosed with SD-OCT, ultra widefield fluorescein angiography, and color fundus photography. The patient received the 0.18-mg FAi OD as first-line treatment for her recurrent uveitis during active inflammation. Two months post-FAi, the patient remained stable and recurrence free.

- VA, CST, and IOP prior to FAi
  - VA: 20/40
  - CST: 309 μm
  - IOP: 24 mmHg
- VA, CST, and IOP 2 months after FAi
  - VA: 20/25
  - CST: 291 μm
  - IOP: 14 mmHg

**Case #7**

A 71-year-old female patient with a medical history of depression, GERD, and vertigo from Meniere’s disease presented with recurrent diffuse NIU-PS OS with late leakage at the macula and optic nerve, which was officially diagnosed in 2021 using SD-OCT, ultra widefield fluorescein angiography, and color fundus photography. The patient’s ocular history included PVD OU and NIU-PS, pars planitis, and CME OS. The patient’s ocular surgeries included cataract surgery with toric PCIOL placement OU in 2017 and an yttrium aluminum garnet (YAG) laser capsulotomy OS in 2018. The patient received sub-tenon triamcinolone (Kenalog) in 2019, but uveitis recurred. In 2021, the dexamethasone 0.7-mg implant was inserted OS, and uveitis recurred 6 months later. The patient received the long-acting 0.18-mg FAi OS on 03/01/2022 during active inflammation and has remained stable and recurrence free for 10 months.

- VA, CST, and IOP prior to FAi
  - VA: 20/80+2
  - CST: 618 μm
  - IOP: 13 mmHg
- VA, CST, and IOP 10 months after FAi
  - VA: 20/50
  - CST: 285 μm
  - IOP: 18 mmHg

**Case #8**

A 72-year-old male patient with a medical history of hypertension presented with recurrent uveitis following ocular surgery OD, diagnosed in 2021. The patient’s ocular history included hypertensive retinopathy OU, a PCIOL placement OD with recurrent NIU-PS and CME (evidenced by OCT, ultra widefield angiography, and fundus exam), and cataract surgery OS. The patient received 2 dexamethasone 0.7-mg implants OD for uveitis recurrence, but the inflammation recurred roughly every 5 months. The patient received the 0.18-mg FAi OD (02/20/2022) during active inflammation and has been stable and recurrence free for 8 months.

- VA, CST, and IOP prior to FAi
  - VA: 20/70
  - CST: 470 μm
  - IOP: 16 mmHg
- VA, CST, and IOP 8 months after FAi
  - VA: 20/60
  - CST: 454 μm
  - IOP: 28 mmHg

**Case #9**

A 75-year-old male patient with a medical history of hypertension, gout, arthritis, polymyalgia, prostate cancer, pulmonary embolism, and post-traumatic stress disorder developed recurrent NIU-PS OD. The patient’s ocular surgical history included PCIOL OU in 2017 and PPV/fluid air exchange/focal laser OD for vitreous hemorrhage secondary to proliferative diabetic retinopathy in 2021. The patient was diagnosed with diffuse NIU-PS with macular edema and late leakage at the optic nerve (imaged via SD‑OCT, ultra widefield fluorescein angiography, and color fundus photography). For initial treatment of uveitis in 2021, the patient received a single dexamethasone 0.7-mg implant OD; uveitis recurred 6 months later, and the patient elected treatment with a long-acting corticosteroid and received the 0.18-mg FAi OD during active inflammation. He has been stable and recurrence free for 9 months.

- VA, CST, and IOP prior to FAi
  - VA: 20/60
  - CST: 284 μm
  - IOP: 8 mmHg
- VA, CST, and IOP 9 months after FAi
  - VA: 20/40
  - CST: 278 μm
  - IOP: 25 mmHg

**Anton M. Kolomeyer, MD, PhD**

**Case #10**

A 74-year-old female patient with a medical history of hypertension, diabetes, hypercholesterolemia, and stroke had been receiving dexamethasone 0.7-mg implant every 3.0 to 5.5 months OU for pars planitis since 2011. The patient had also received posterior sub-tenon triamcinolone (Kenalog) OU previously.

The patient’s ocular history included moderate nonproliferative diabetic retinopathy, intermediate age-related macular degeneration, pseudophakia, ocular hypertension, and PVD OU. OCT, fluorescein angiography, and color fundus photography were used in the diagnostic work-up. Upon examination, the patient had vitreous snowballs, anterior chamber cells, and macular edema. The patient received a short-acting IVI steroid a few weeks prior, then received 0.18-mg FAi OD on 1/17/23 during active inflammation, then another implant OS on 2/7/23 to treat vitritis and CME. After the FAis OU, the patient has remained recurrence free and stable for more than 4 months.

- OS
  - VA, CST, and IOP prior to FAi
    - VA: 20/200
    - CST: 194 μm
    - IOP: 12 mmHg
  - VA, CST, and IOP 5 months after FAi
    - VA: 20/40
    - CST: 194 μm
    - IOP: 13 mmHg
- OD
  - VA, CST, and IOP prior to FAi
    - VA: 20/30
    - CST: 211 μm
    - IOP: 12 mmHg
  - VA, CST, and IOP 4 months after FAi
    - VA: 20/40
    - CFT: 204 μm
    - IOP: 14 mmHg

**Case #11**

An 87-year-old male patient with a medical history of type 2 diabetes, hypertension, hypercholesterolemia, and benign prostate hyperplasia had been receiving dexamethasone 0.7-mg implant every 3 to 5 months OU (OS since 2012, OD since 2013) for treatment of panuveitis, with prior IVI triamcinolone (Triescence) OU as well. The patient was pseudophakic and had an ocular history of POAG OU and PVD OU. The patient’s ocular surgical history included an epiretinal membrane peel (OD, 2010) and tube shunt implantation (OD, 2010; OS, 2017). The physician used OCT, fluorescein angiography, and color fundus photography as part of the uveitis diagnostic work-up. Upon examination, the patient had vitritis, anterior chamber cells, and macular edema. The patient received a short-acting IVI steroid during active inflammation to treat vitritis and CME a few weeks prior to the 0.18-mg FAi OU (OD 08/23/2019, OS 08/02/2019).

The patient was recurrence free OU for the anticipated duration of the implants. At about 40 months post-FAi, recurrence was treated with dexamethasone 0.7 mg implant OU, followed by a second FAi several weeks later. The patient has remained recurrence free and stable OU for at least 2 months after the second FAi.

- OS
  - VA, CST, and IOP when the patient received his first FAi
    - VA: 20/40
    - CST: 161 μm
    - IOP: 17 mmHg
  - VA, CST, and IOP 40 months after the patient received his first FAi; 20^th^ injection of dexamethasone
    - VA: 20/50
    - CST: 227 μm
    - IOP: 15 mmHg
  - VA, CST, and IOP 2.5 months after the patient received his second FAi
    - VA: 20/100
    - CST: 232 μm
    - IOP: 11 mmHg
- OD
  - VA, CST, and IOP when the patient received his first FAi
    - VA: 20/80
    - CST: 184 μm
    - IOP: 24 mmHg
  - VA, CST, and IOP 40 months after the patient received his first FAi; 20^th^ injection of dexamethasone
    - VA: 20/100
    - CST: 218 μm
    - IOP 8 mmHg
  - VA, CST, and IOP 2.5 months after the patient received his second FAi
    - VA: 20/150
    - CST: 219 μm
    - IOP: 7 mmHg

**David Eichenbaum, MD**

**Case #12**

A 68-year-old female patient with a medical history of diet-controlled hyperlipidemia had received several ocular surgeries OS, including cataract extraction and intraocular lens (IOL) placement, PPV, and pars plana lensectomy. In August of 2019, the patient had a dislocated IOL and received a PPV IOL exchange Gore-Tex sutured Akreos AO-60 placed OS. Eight weeks later, she complained of eye pain and worsening vision with 1+ anterior chamber and vitreous cell flare, VA of 20/125, and 17 mmHg IOP, but the sutured IOL remained stable and well-centered.

Based on the clinical exam showing posterior cells and OCT images demonstrating macular edema, this patient was diagnosed with uveitic CME OS on 10/1/2019. She started preservative-free artificial tears twice daily (BID) and an intravitreal triamcinolone (Triesence) injection. Initially the short-term corticosteroids helped, but 4 months later, the vision loss, pain, and CME recurred. A second triamcinolone (Triesence) IVI was given in February of 2020, which helped with her pain, but her vision was still blurry 7 weeks post-injection.

Due to her persistent uveitic CME, the 0.18-mg FAi was administered on 4/3/2020; prior to the FAi administration, a short-acting IVI steroid was given to the patient to quiet the eye. The patient developed a persistent steroid response to the FAi 3 months following insertion; thus, the patient was started on topical brimonidine and brinzolamide ophthalmic suspension BID and has regained a stable IOP. Thirty-three months following the FAi, OS remained stable and pain-free.

- Previous VA, CST, and IOP prior to FAi
  - VA: 20/125
  - CST: 510 µm
  - IOP: 14 mmHg
- Most recent VA, CST, and IOP 33 months after FAi
  - VA: 20/40+
  - CST: 247 µm
  - IOP: 15 mmHg (maintained on brimonidine 0.1% and brinzolamide ophthalmic suspension, both BID)

**John W. Kitchens, MD**

**Case #13**

A 74-year-old male patient underwent complicated cataract extraction OS on 10/21/20 with zonular loss (no retained lens fragments) and anterior chamber paracentesis for elevated IOP. The patient experienced recurrent inflammation, rare cell presence in the vitreous (based on clinical exam findings), and presence of edema on imaging. The patient was diagnosed with postoperative intermediate uveitis. The patient’s CME was treated with topical difluprednate, as well as steroid-response ocular hypertension managed with topical timolol and brimonidine eye drops.

During his initial visit with the retina specialist on 2/21/2022, the patient had a dislocated IOL and recurrent CME. An anterior chamber IOL exchange was performed on 05/10/2022, but it did not prevent recurrent edema and intermediate uveitis. Two months later, his VA was 20/40, IOP 24 mmHg, and CST 379 μm. Due to the patient’s recurrent uveitis, an 0.18-mg FAi implant was inserted two weeks later during active inflammation. However, after 7 weeks, initial response to the FAi was limited, so dexamethasone 0.7-mg implant was given. Seven months post-FAi, OS has remained stable and recurrence free.

- VA, CST, and IOP prior to FAi
  - VA: 20/40
  - CST: 379 µm
  - IOP: 24 mmHg
- VA, CST, and IOP 7 months after FAi
  - VA: 20/30
  - CST: 271 µm
  - IOP: 20 mmHg

**Case #14**

A 63-year-old female patient underwent multiple ocular surgeries OS, including uncomplicated buckle and vitrectomy with laser and gas for macula-off retinal detachment repair in November and December 2020. The patient experienced chronic inflammation post-surgery and was diagnosed with intermediate uveitis with recurrent CME based on clinical exam, the presence of rare cells in the vitreous, and macular edema on OCT. The patient received two IVI triamcinolone (Triesence) injections OS (02/08/2021 and 04/26/2021) to treat intermediate uveitis and CME with epiretinal membrane.

On 10/18/2021, the patient underwent a cataract extraction and IOL placement along with a vitrectomy and membrane peel. Eight months after retinal detachment surgery, and 10 weeks after the second intravitreal injection, the patient’s VA was 3/200 and CST 398 μm. The patient experienced recurrent uveitic CME every 3 to 4 months. The 0.18-mg FAi was initiated 4.5 months after the last dexamethasone injection (during active inflammation), and the patient has remained recurrence free through 6 months of follow up.

- VA, CST, and IOP prior to FAi
  - VA: 3/200
  - CST: 398 µm
  - IOP: 10 mmHg
- VA, CST, and IOP 6 months after FAi
  - VA: 20/60
  - CST: 265 µm
  - IOP: 19 mmHg

**Case #15**

A 46-year-old male patient presented with CME OS in 2004 and received sub-tenon triamcinolone (Triesence) injections twice. The patient underwent cataract surgery OS in 2005. In 2012, he developed CME OD, was formally diagnosed with intermediate uveitis (based on clinical exam, OCT angiography, and OCT) and developed non-arteritic anterior ischemic optic neuropathy (NAION) OS. He was suspected of having multiple sclerosis. The patient had macular edema (CST 409 μm OD, 337 μm OS) and declining vison (VA 20/60 OD, 20/40 OS).

Due to recurrent intermediate uveitis and uveitic CME, the patient received IVI triamcinolone (Triesence) injections OU every 3 to 4 months. In 2016, he developed cataract OD and underwent cataract extraction. That same year, systemic methotrexate was initiated, which helped to decrease his injection frequency to every 6 to 7 months, but the methotrexate was discontinued in 2018 due to *C. difficile* colitis. In 2018, the patient also developed NAION OD. In 2019, sustained-release dexamethasone implants were administered OU and extended therapy to every 6 months.

Due to the frequency of injections, the long-acting 0.18-mg FAi was selected for therapy (OS 12/17/2021, OD 1/18/2022) and given during active inflammation. The FAi injection OD was complicated by presumed endophthalmitis, so a vitreous tap and antibiotic injection (vancomycin and ceftazidime) was performed, even though Gram stain and cultures were negative. One year after FAi administrations OU, the patient remained recurrence-free and stable.

- OD
  - VA, CST, and IOP prior to FAi
    - VA: 20/30
    - CST: 240 µm
    - IOP: 11 mmHg
  - VA, CST, and IOP 12 months after FAi
    - VA: 20/30
    - CST: 247 µm
    - IOP: 9 mmHg
- OS
  - VA, CST, and IOP prior to FAi
    - VA: 20/30
    - CST: 252 µm
    - IOP: 14 mmHg
- VA, CST, and IOP 12 months after FAi
  - VA: 20/25
  - CST: 241 µm
  - IOP: 12 mmHg

Abbreviations

BID twice daily

CME cystoid macular edema

CST central subfield thickness

GERD gastroesophageal reflux

FAi fluocinolone acetonide implant

ILM internal limiting membrane

IOL intraocular lens

IOP intraocular pressure

IVI intravitreal injection

NAION non-arteritic anterior ischemic optic neuropathy

NIU-PS non-infectious uveitis affecting the posterior segment

NSAID nonsteroidal anti-inflammatory drug

OCT optical coherence tomography

OD right eye

OS left eye

OU both eyes

PCIOL posterior chamber intraocular lenses

POAG primary open angle glaucoma

PPV pars plana vitrectomy

PVD posterior vitreous detachment

SD-OCT spectral domain optical coherence tomography

VA visual acuity

VEGF vascular endothelial growth factor

YAG yttrium aluminum garnet
